# Supplementary material for: Differential expression of miR-1, a putative tumor suppressing microRNA, in cancer resistant and cancer susceptible mice
Source: PeerJ. 2013 Apr 16;1:e68. doi: 10.7717/peerj.68 (PMC3642704; doi:10.7717/peerj.68)
Supplement: Figure S3 — Apoptosis in miR-1 or scrambled control miR transfected cells (SC) was measured at 48, 72 and 96 h by AnnexinV and PI staining measured by FACS analysis. Shown is a representative sample at 72 h post-transfection for the A5 (A) SC and (B) miR-1 transfected cells and for the B9 (C) SC and (D) miR-1 transfected cells. The gating of the cells and the percentage of apoptotic cells staining positive for AnnexinV, propridium iodine or both (upper right quadrant) are shown. [file peerj-01-68-s003.pdf]

A. 72 hour-post transfection of Scrambled Control miR in A5 Cells

Apoptosis: Annexin and PI staining

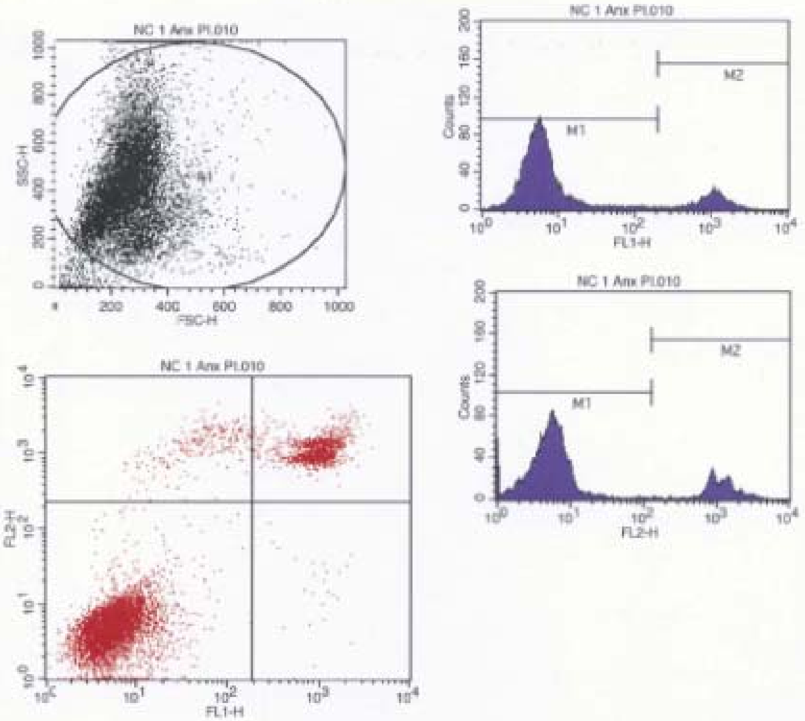

Quadrant Statistics

File: NC 1 Anx PI.010
Sample ID: NC 1 Anx PI
Tube: Untitled
Acquisition Date: 29-Jan-13
Gated Events: 10000
X Parameter: FL1-H (Log)
Quad Location: 164, 219

Log Data Units: Linear Values
Patient ID:
Panel: Untitled Acquisition Tube List
Gate: G1
Total Events: 10529
Y Parameter: FL2-H (Log)

Quad	Events	% Gated	% Total	X Mean	X Geo Mean	Y Mean	Y Geo Mean
UL	248	2.48	2.36	67.42	51.75	1442.54	1272.11
UR	1334	13.34	12.67	1039.51	944.37	1140.61	1075.84
LL	8394	83.94	79.72	6.42	5.61	6.04	5.06
LR	24	0.24	0.23	1072.52	916.04	24.96	15.07

B. 72 hour-post transfection of *miR-1* in A5 Cells

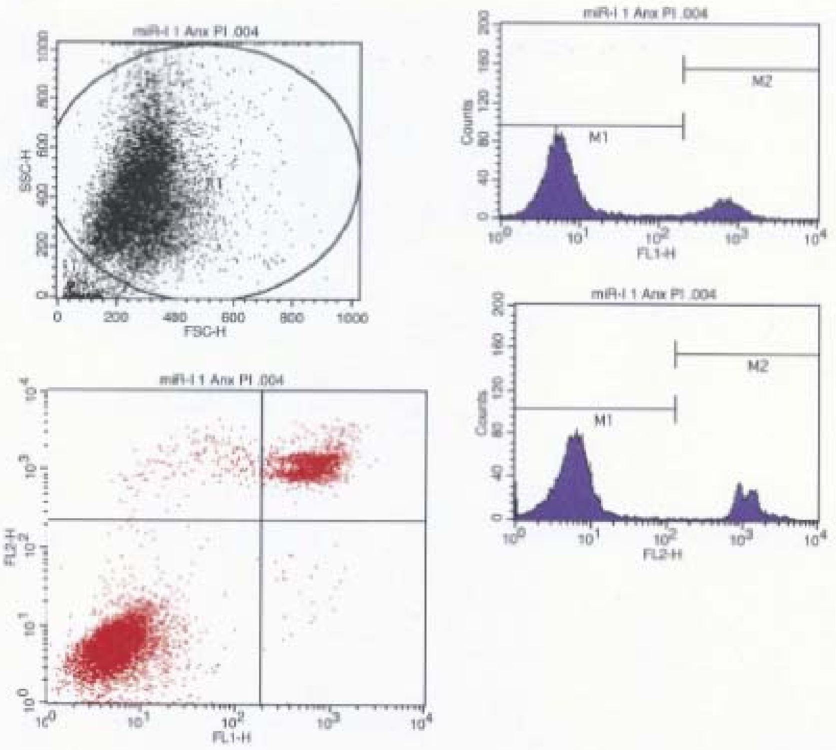

Quadrant Statistics

File: miR-I 1 Anx PI .004
Sample ID: miR-I 1 Anx PI
Tube: Untitled
Acquisition Date: 29-Jan-13
Gated Events: 10000
X Parameter: FL1-H (Log)
Quad Location: 184, 219

Log Data Units: Linear Values
Patient ID:
Panel: Untitled Acquisition Tube List
Gate: G1
Total Events: 10666
Y Parameter: FL2-H (Log)

Quad	Events	% Gated	% Total	X Mean	X Geo Mean	Y Mean	Y Geo Mean
UL	188	1.88	1.76	65.16	44.88	1427.97	1255.45
UR	1636	16.36	15.34	678.36	614.50	1174.88	1106.38
LL	8152	81.52	76.43	6.26	5.51	6.60	5.66
LR	24	0.24	0.23	611.84	519.78	34.86	23.85

C. 72 hour-post transfection of *SC* in B9 Cells

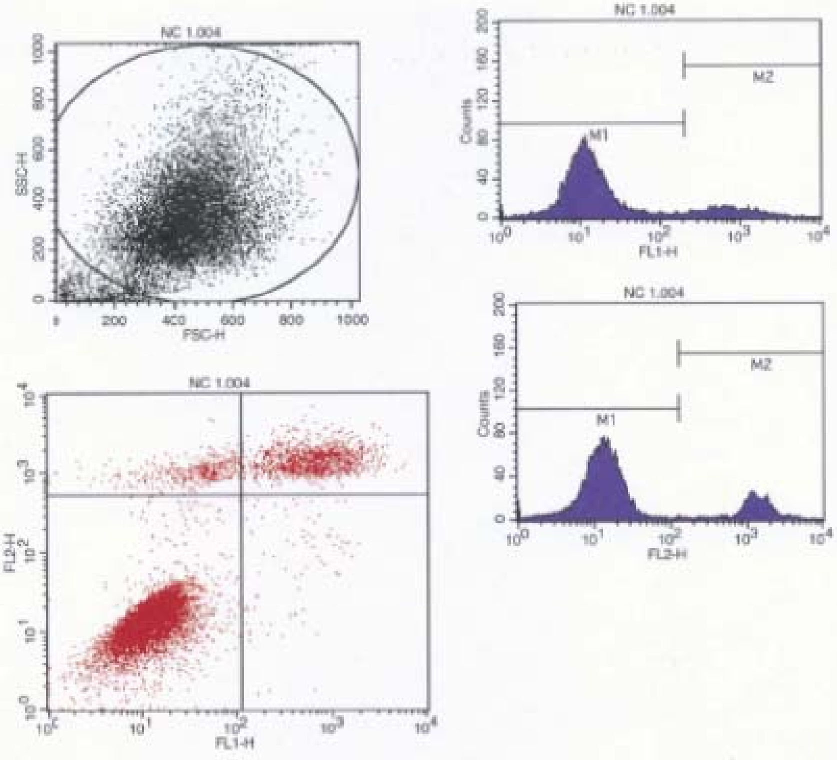

Quadrant Statistics

File: NC 1.004
Sample ID: NC 1
Tube: Untitled
Acquisition Date: 13-Feb-13
Gated Events: 10080
X Parameter: FL1-H (Log)
Quad Location: 109, 514

Log Data Units: Linear Values
Patient ID:
Panel: Untitled Acquisition Tube List
Gate: G1
Total Events: 10948
Y Parameter: FL2-H (Log)

Quad	Events	% Gated	% Total	X Mean	X Geo Mean	Y Mean	Y Geo Mean
UL	410	4.10	3.74	44.64	34.30	1099.65	1048.34
UR	1184	11.84	10.81	821.60	633.25	1504.71	1419.98
LL	8330	83.30	76.09	13.18	11.43	15.73	12.54
LR	76	0.76	0.69	581.52	414.08	140.71	68.73

Page 1

D. 72 hour post-transfection of *miR-1* in B9 cells

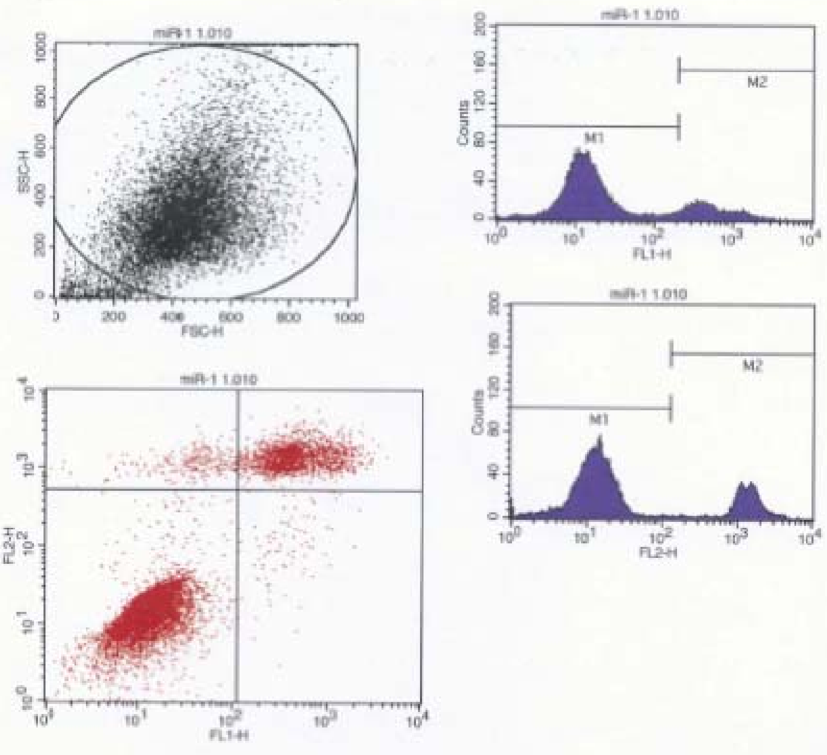

Quadrant Statistics

File: miR-1 1.010
Sample ID: miR-1 1
Tube: Untitled
Acquisition Date: 13-Feb-13
Gated Events: 1000C
X Parameter: FL1-H (Log)
Quad Location: 109, 514

Log Data Units: Linear Values
Patient ID:
Panel: Untitled Acquisition Tube List
Gate: G1
Total Events: 10950
Y Parameter: FL2-H (Log)

Quad	Events	% Gated	% Total	X Mean	X Geo Mean	Y Mean	Y Geo Mean
UL	258	2.58	2.36	46.05	35.30	1212.31	1151.73
UR	1965	19.65	17.95	575.99	458.38	1440.97	1367.86
LL	7688	76.88	70.21	14.42	12.50	15.53	12.41
LR	89	0.89	0.81	486.89	370.86	150.35	90.66
