# Supplementary material for: Differential expression of miR-1, a putative tumor suppressing microRNA, in cancer resistant and cancer susceptible mice
Source: PeerJ. 2013 Apr 16;1:e68. doi: 10.7717/peerj.68 (PMC3642704; doi:10.7717/peerj.68)
Supplement: Figure S4 — Cell cycle parameters for scrambled control miRNA (SC) and miR-1 transfected cells were measured for A5 cells at (A) 48 h, (B), 72 h and (C) 96 h and for B9 cells at (D) 48 h, (E) 72 h, and (F) 96 h by staining with propidium iodide and sorting via flow cytometry. The percentage of gated cells for G0-G1, S and G2-M phases are indicated. [file peerj-01-68-s004.pdf]

A. 96 hour-post transfection of SC in A5 Cells

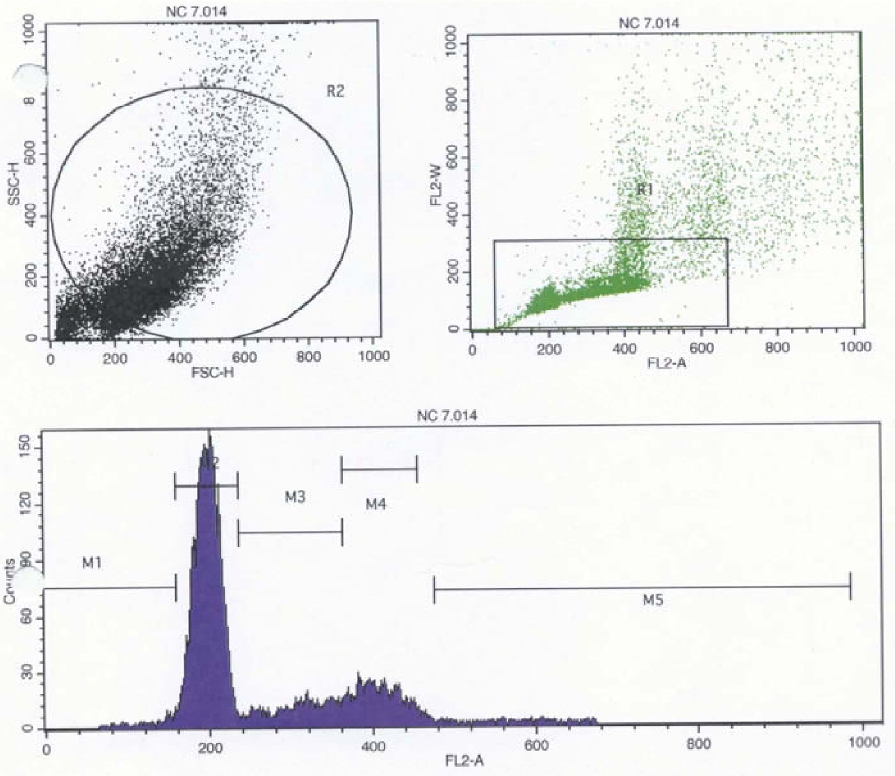

Histogram Statistics

File: NC 7.014
Sample ID: NC 7
Tube: Untitled
Acquisition Date: 12-Feb-13
Gated Events: 8663
X Parameter: FL2-A (Linear)

Log Data Units: Linear Values
Patient ID:
Panel: Untitled Acquisition Tube List
Gate: G3
Total Events: 15317

Marker	Left, Right	Events	% Gated	% Total	Mean	Geo Mean	CV	Median	Peak Ch
All	0, 1023	8663	100.00	56.56	257.97	242.03	39.23	207.00	201
M1	0, 159	148	1.71	0.97	131.96	128.95	19.83	143.00	158
M2	159, 236	5651	65.23	36.89	197.23	196.70	7.28	198.00	201
M3	236, 362	1117	12.89	7.29	308.79	306.73	11.36	313.00	317
M4	362, 455	1447	16.70	9.45	405.43	404.68	6.09	404.00	380
M5	475, 987	246	2.84	1.61	569.13	566.40	9.79	570.00	483

9uH A5 2-12-13

B. 96 hour-post transfection of *miR-1* in A5 Cells

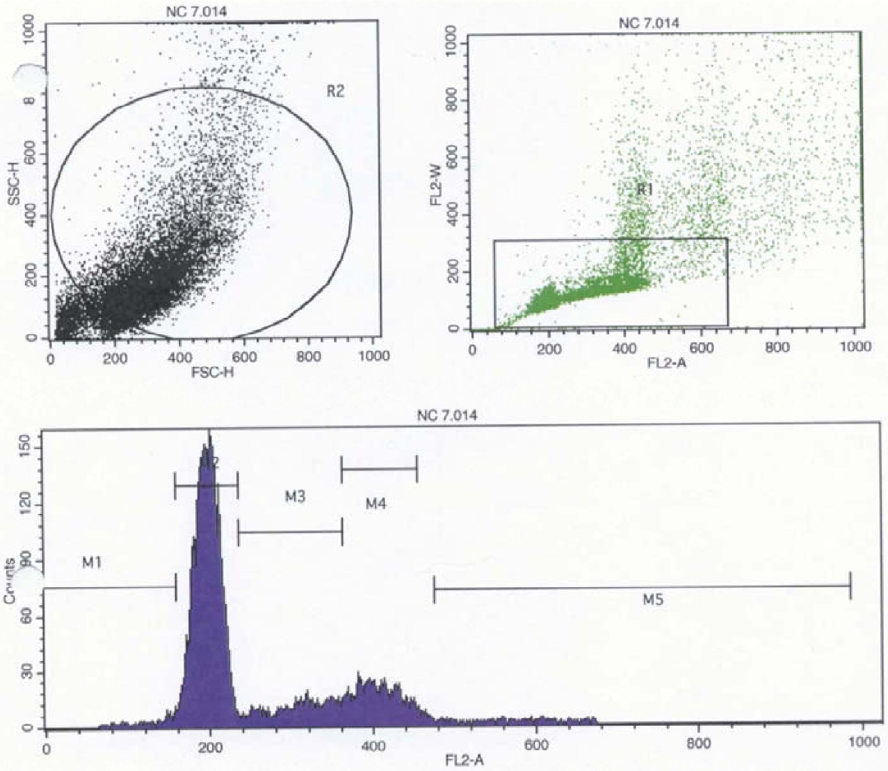

Histogram Statistics

File: NC 7.014
Sample ID: NC 7
Tube: Untitled
Acquisition Date: 12-Feb-13
Gated Events: 8663
X Parameter: FL2-A (Linear)

Log Data Units: Linear Values
Patient ID:
Panel: Untitled Acquisition Tube List
Gate: G3
Total Events: 15317

Marker	Left, Right	Events	% Gated	% Total	Mean	Geo Mean	CV	Median	Peak Ch
All	0, 1023	8663	100.00	56.56	257.97	242.03	39.23	207.00	201
M1	0, 159	148	1.71	0.97	131.96	128.95	19.83	143.00	158
M2	159, 236	5651	65.23	36.89	197.23	196.70	7.28	198.00	201
M3	236, 362	1117	12.89	7.29	308.79	306.73	11.36	313.00	317
M4	362, 455	1447	16.70	9.45	405.43	404.68	6.09	404.00	380
M5	475, 987	246	2.84	1.61	569.13	566.40	9.79	570.00	483

Page

96H A5 2-12-13

C. 96 hour-post transfection of *SC* in B9 Cells

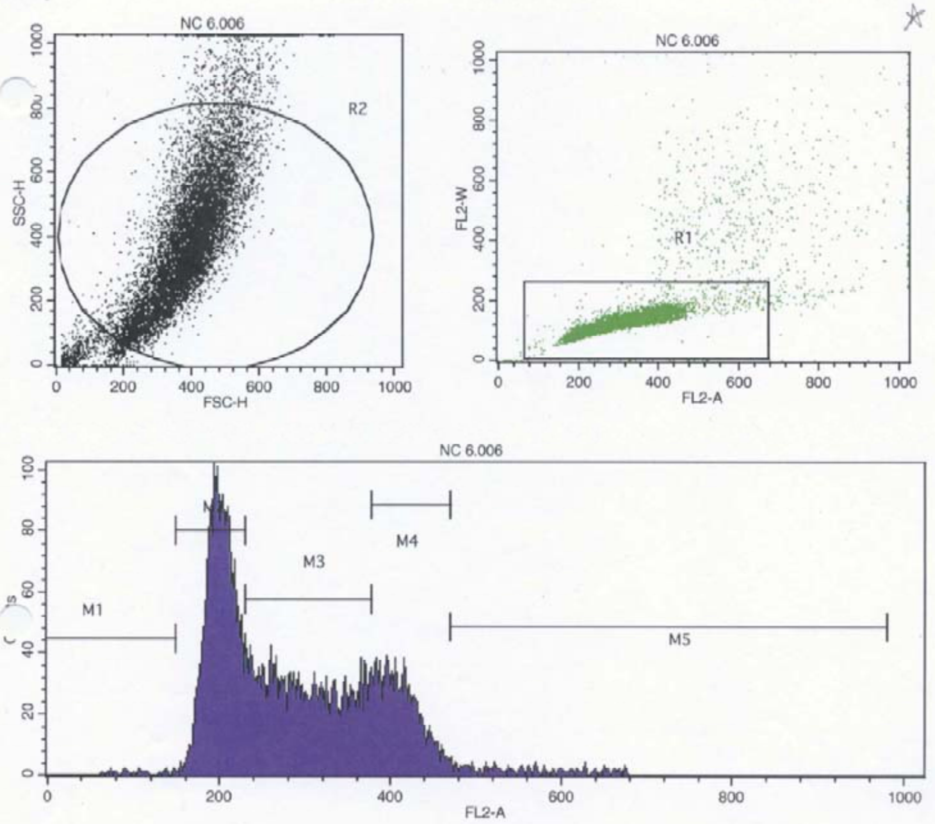

Histogram Statistics

File: NC 6.006
Samɔle ID: NC 6
Tube: Untitled
Acquisition Date: 26-Feb-13
Gated Events: 9535
X Parameter: FL2-A (Linear)

Log Data Units: Linear Values
Patient ID:
Panel: Untitled Acquisition Tube List
Gate: G3
Total Events: 11726

Marker	Left, Right	Events	% Gated	% Total	Mean	Geo Mean	CV	Median	Peak Ch
All	0, 1023	9535	100.00	81.32	287.48	273.86	31.85	266.00	193
M1	0, 149	31	0.33	0.26	107.06	104.04	24.08	108.00	76
M2	149, 230	3648	38.26	31.11	200.53	199.94	7.61	201.00	193
M3	230, 377	3906	40.96	33.31	299.99	296.80	14.56	297.00	235
M4	377, 469	1823	19.12	15.55	410.42	409.81	5.49	408.00	394
M5	469, 981	197	2.07	1.68	551.52	548.03	11.38	541.00	470

D. 96 hour post-transfection of *miR-1* in B9 cells

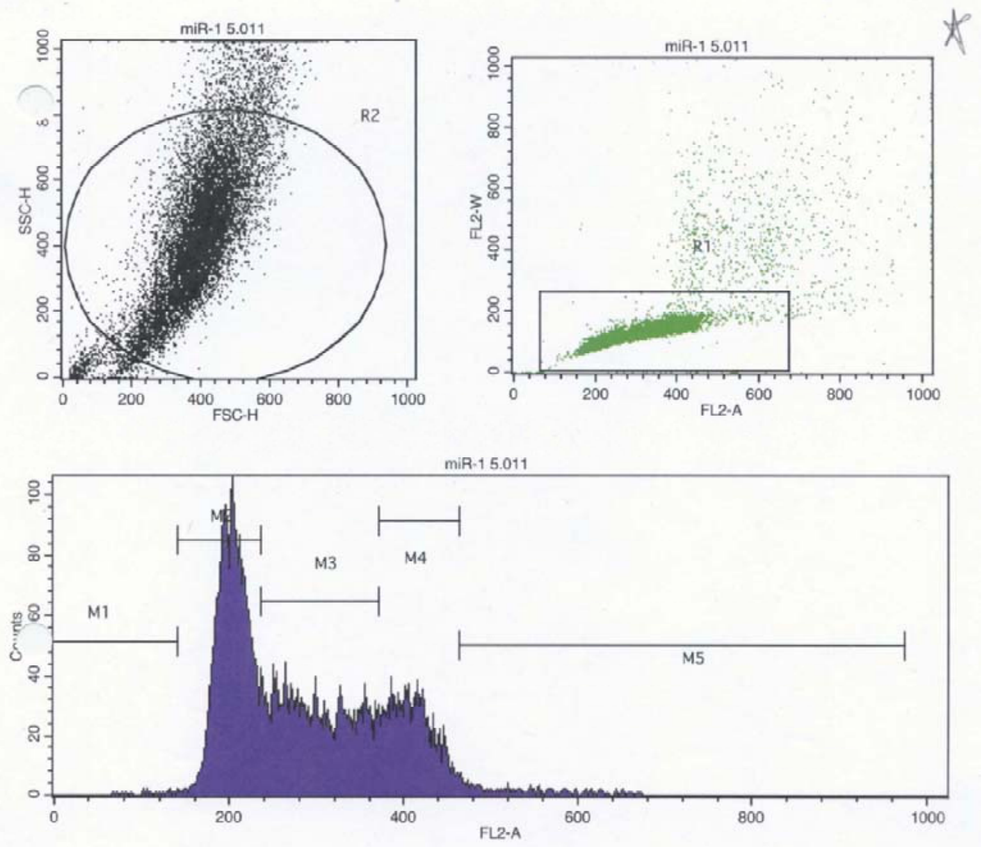

Histogram Statistics

File: miR-1 5.011
Sample ID: miR-1 5
Tube: Untitled
Acquisition Date: 26-Feb-13
Gated Events: 9395
X Parameter: FL2-A (Linear)

Log Data Units: Linear Values
Patient ID:
Panel: Untitled Acquisition Tube List
Gate: G3
Total Events: 12329

Marker	Left, Right	Events	% Gated	% Total	Mean	Geo Mean	CV	Median	Peak Ch
All	0, 1023	9395	100.00	76.20	287.25	274.08	31.26	264.00	204
M1	0, 141	19	0.20	0.15	108.00	105.71	20.43	108.00	104
M2	141, 236	3853	41.01	31.25	203.60	202.92	8.14	204.00	204
M3	236, 371	3454	36.76	28.02	300.05	297.36	13.37	298.00	264
M4	371, 463	1957	20.83	15.87	408.29	407.65	5.64	406.00	385
M5	463, 975	186	1.98	1.51	530.62	527.50	11.09	516.50	464
