# Supplementary material for: Differential expression of miR-1, a putative tumor suppressing microRNA, in cancer resistant and cancer susceptible mice
Source: PeerJ. 2013 Apr 16;1:e68. doi: 10.7717/peerj.68 (PMC3642704; doi:10.7717/peerj.68)
Supplement: Table S2 — Genes predicted to be targets of the validated five miRNAs. [file peerj-01-68-s007.docx]

| **miR-1 Predicted Targets** |  |  |
| --- | --- | --- |
| 1110003E01Rik | Slc7a6 |  |
| Ankrd13c | Smarcd1 |  |
| Arcn1 | Sri |  |
| Bag4 | Ss18 |  |
| Bcl2 | Syt1 |  |
| Bdnf | Tbc1d15 |  |
| Cap1 | Timp3 |  |
| Cdk14 | Trim2 |  |
| Cltc | Twf1 |  |
| Cmpk2 | Ust |  |
| Col4a3 | Zfp148 |  |
| Coro1c | Zfp280d |  |
| Dll1 | Zfp36L1 |  |
| Edn1 | Zfp36L2 |  |
| Eif4e |  |  |
| Entpd7 |  |  |
| Erc1 |  |  |
| Ets1 |  |  |
| Faf2 |  |  |
| Foxp1 |  |  |
| G6pdx |  |  |
| Gja1 |  |  |
| Hand2 |  |  |
| Hdac4 |  |  |
| Hsp90b1 |  |  |
| Kdsr |  |  |
| Klf13 |  |  |
| Lasp1 |  |  |
| Mapk1 |  |  |
| Mapk3 |  |  |
| Mef2c |  |  |
| Met |  |  |
| Mmd |  |  |
| Mtap1a |  |  |
| Pdik1l |  |  |
| Pik3c2a |  |  |
| Pim1 |  |  |
| Plxna4 |  |  |
| Prkce |  |  |
| Ptpn14 |  |  |
| Rfesd |  |  |
| Rrbp1 |  |  |
| Rufy2 |  |  |
| Serp1 |  |  |
| Slc39a10 |  |  |
| **miR-124 Predicted Targets** |  |  |
| 4933426M11Rik | Sox2 | |
| Abcc1 | Sox9 | |
| Apln | Trib3 | |
| B4galt1 | Ttl | |
| Bmp6 | Vamp3 | |
| Cd164 | Vim | |
| Cdk6 | Xpo4 | |
| Cebpa |  |  |
| Chic1 |  |  |
| Ctns |  |  |
| Ddx3x |  |  |
| Efnb1 |  |  |
| Eya4 |  |  |
| Ezh2 |  |  |
| Flot2 |  |  |
| Gga2 |  |  |
| Hes1 |  |  |
| Igfbp7 |  |  |
| Itgb1 |  |  |
| Itpr3 |  |  |
| Klhl24 |  |  |
| Lcp1 |  |  |
| Lhx2 |  |  |
| Limk1 |  |  |
| Lpp |  |  |
| Lrrc58 |  |  |
| Magt1 |  |  |
| Mecom |  |  |
| Nfatc1 |  |  |
| Nipa1 |  |  |
| Pabpc4L |  |  |
| Pik3c2a |  |  |
| Plxna3 |  |  |
| Prrx1 |  |  |
| Prtg |  |  |
| Ptbp2 |  |  |
| Ptpn12 |  |  |
| Ptprj |  |  |
| Rhog |  |  |
| Rnpepl1 |  |  |
| Rock2 |  |  |
| Rod1 |  |  |
| Sept10 |  |  |
| Slc16a1 |  |  |
| Slitrk6 |  |  |
| \| **mir-133a Predicted Targets** \| \| --- \| \| Btbd3 \| \| Capn5 \| \| Casp9 \| \| Ctgf \| \| Fscn1 \| \| Gnrhr \| \| Hspa4 \| \| Hspd1 \| \| Klf15 \| \| Krt7 \| \| Krt8 \| \| Lasp1 \| \| Myh9 \| \| Ppp3cb \| \| Slc24a4 \| \| Smarcd1 \| \| Srf \| \| Tpm4 \| \| Twf1 \| \| **miR-134 Predicted Targets** \| \| Abcc1 \| \| Bard1 \| \| Cdk6 \| \| Creb1 \| \| Foxa2 \| \| Ghr \| \| Gm608 \| \| Grik3 \| \| Lhx2 \| \| Limk1 \| \| Mtpn \| \| Nr5a2 \| \| Ppt1 \| \| Ptpn2 \| \| Rab27a \| \| Sox2 \| |  |  |
|  |  |  |
|  |  |  |
|  |  |  |
|  |  |  |
|  |  |  |
|  |  |  |
| **miR-192 Predicted Targets** |  |  |
| Arhgap19 |  |  |
| Cdc7 |  |  |
| Cxcl2 |  |  |
| Dhfr |  |  |
| Ercc3 |  |  |
| Ercc4 |  |  |
| Gmeb1 |  |  |
| Mdm2 |  |  |
| Pabpc4 |  |  |
| Per1 |  |  |
| Per2 |  |  |
| Per3 |  |  |
| Ptprt |  |  |
| Rb1 |  |  |
| Rsad2 |  |  |
| Sip1 |  |  |
| Slc5a3 |  |  |
| Tgfb1 |  |  |
| Wnk1 |  |  |
| Xiap |  |  |
| Zeb1  Zeb2 |  |  |

| \| **miR-206 Predicted Targets** \| \| --- \| \| Adar \| \| Aftph \| \| Bach2 \| \| Bdnf \| \| Bsn \| \| Cap1 \| \| Celf4 \| \| Cmpk1 \| \| Coro1c \| \| Cplx2 \| \| Edem1 \| \| Eif4E \| \| Esr1 \| \| Ets1 \| \| Fgfbp1 \| \| Foxp1 \| \| Fstl1 \| \| Gabpb2 \| \| Gja1 \| \| Hdac4 \| \| Hsp90B1 \| \| Lass4 \| \| Lhfp \| \| Met \| \| Mmd \| \| Nfat5 \| \| Notch3 \| \| Pax3 \| \| Pola1 \| \| Ptplad1 \| \| Ptprj \| \| Ptprk \| \| Ptprz1 \| \| Rb1cc1 \| \| Sgms2 \| \| Slc44a1 \| \| Smarcd1 \| \| Tnks2 \| \| Tspyl4 \| \| Unc119B \| \| Utrn \| \| Vamp2 \| \| Zxdb \| |
| --- | --- | --- | --- | --- | --- | --- | --- | --- | --- | --- | --- | --- | --- | --- | --- | --- | --- | --- | --- | --- | --- | --- | --- | --- | --- | --- | --- | --- | --- | --- | --- | --- | --- | --- | --- | --- | --- | --- | --- | --- | --- | --- | --- | --- |
